# Posttraumatic Stress Disorder, Adverse Childhood Events, and Buccal Cell Telomere Length in Elderly Swiss Former Indentured Child Laborers

**DOI:** 10.3389/fpsyt.2016.00147

**Published:** 2016-08-31

**Authors:** Andreas Lorenz Küffer, Aoife O’Donovan, Andrea Burri, Andreas Maercker

**Affiliations:** ^1^Department of Psychology, Division for Psychopathology and Clinical Intervention, University of Zurich, Zurich, Switzerland; ^2^University Research Priority Program “Dynamics of Healthy Aging”, University of Zurich, Zurich, Switzerland; ^3^Department of Psychiatry, University of California in San Francisco, San Francisco, CA, USA; ^4^San Francisco Veterans Affairs Medical Center, San Francisco, CA, USA; ^5^Health and Rehabilitation Research Institute, Auckland University of Technology, Auckland, New Zealand; ^6^Waitemata Pain Service, Department of Anaesthesia and Perioperative Medicine, North Shore Hospital, Auckland, New Zealand

**Keywords:** PTSD, adverse childhood events, old adults, buccal cell telomere length, qPCR, Swiss indentured child laborers

## Abstract

Posttraumatic stress disorder (PTSD) is associated with increased risk for age-related diseases and early mortality. Accelerated biological aging could contribute to this elevated risk. The aim of the present study was to assess buccal cell telomere length (BTL) – a proposed marker of biological age – in men and women with and without PTSD. The role of childhood trauma was assessed as a potential additional risk factor for shorter telomere length. The sample included 62 former indentured Swiss child laborers (age: M = 76.19, SD = 6.18) and 58 healthy controls (age: M = 71.85, SD = 5.97). Structured clinical interviews were conducted to screen for PTSD and other psychiatric disorders. The Childhood Trauma Questionnaire (CTQ) was used to assess childhood trauma exposure. Quantitative polymerase chain reaction was used to measure BTL. Covariates include age, sex, years of education, self-evaluated financial situation, depression, and mental and physical functioning. Forty-eight (77.42%) of the former indentured child laborers screened positive for childhood trauma, and 21 (33.87%) had partial or full-blown PTSD. Results did not support our hypotheses that PTSD and childhood trauma would be associated with shorter BTL. In fact, results revealed a trend toward longer BTL in participants with partial or full PTSD [*F*(2,109) = 3.27, *p* = 0.04, η^2^ = 0.06], and longer BTL was marginally associated with higher CTQ scores (age adjusted: β = 0.17 [95% CI: −0.01 to 0.35], *t* = 1.90, *p* = 0.06). Furthermore, within-group analyses indicated no significant association between BTL and CTQ scores. To the best of our knowledge, this is the first study exploring the association between childhood trauma and BTL in older individuals with and without PTSD. Contrary to predictions, there were no significant differences in BTL between participants with and without PTSD in our adjusted analyses, and childhood adversity was not associated with BTL. Possible explanations and future research possibilities are discussed.

## Introduction

Until the late 1970s, it was a common practice in Switzerland to have orphans, children of single-parent families, or even children from divorced or separated parents removed by the authorities from their family environment into foster care. Unlike typical foster care, the children were specifically sent into farmers’ homes or other types of working class families in rural areas to work. Nowadays, this would essentially be described as indentured servitude and would clearly be classified as child labor. Historic studies have documented the harsh environment in which these individuals grew up, reporting that a large proportion of the children were regularly beaten, emotionally and sexually abused, and that some of them died or were murdered (1). Most of these Swiss indentured child laborers [in German: “Verdingkinder”] are now in late life, and studies have reported high prevalence of adverse childhood experiences and poor mental health (2, 3).

Telomere length (TL) has been proposed as one of the mechanisms of early life stress effects on physical health (4). Telomeres are involved in genome stability and the regulation of cellular proliferation. TL varies between and within species, and within subjects over time, as well as between different cell populations (5). Telomeres shorten with each mitotic division, because of the inability to fully replicate the 3′-end of the DNA strand (6). Telomeres have also been shown to shorten exogenous sources (e.g., UV-light, radiation, ozone, etc.), oxidative stress, and/or inflammation (7, 8). A considerable number of studies – including experimental animal models – have found significant relationships between stress, traumatic stress (and its consequences), such as posttraumatic stress disorder (PTSD) and TL [e.g., Ref. (9–12)].

Further studies have now linked adverse childhood events (ACEs) with TL. A study on 43 patients with PTSD (including 18 PTSD patients with multiple categories with of childhood trauma), and 47 control subjects found significantly shorter leukocyte TL (LTL) in participants with PTSD compared with control subjects, only if the participants with PTSD also suffered from exposure to multiple categories of childhood trauma (13). In 2014, for example, the prospective longitudinal Dunedin Study conducted on *n* = 1,037 subjects 11 years of age showed that persistent internalizing disorders in men – but not in women – predicted LTL at 27 years later in a dose–response manner (i.e., the more years they reported to have suffered, the shorter were their LTL) (14). In another cross-sectional study by Drury and colleagues including 80 children (age 5–15 years) exposed to family violence, the authors found that the number of adverse life events was associated with shorter buccal cell TL (BTL) (15). Savolainen et al. studied a sample of 1,486 adults (mean age of 61.5 years) at the time of tissue collection (16). They assessed separation from parents in childhood (*n* = 215), as well as self-reported physically and emotionally traumatic experiences throughout the lifespan. Even though they did not find a significant association between LTL and either early life parental separation or emotional and physical trauma across the full sample, they found shorter LTL in the participants who were separated from their parents during childhood and who also reported traumatic experiences. A study of a sample of 3,000 subjects, PTSD alone was associated with shorter LTL (17). One study that investigated LTL in 496 elderly individuals (mean age of 70.6 years; SD = 7.4 years) found no significant association with childhood abuse, recent negative life events, or loneliness (18). Only early separation from parents was marginally negatively associated with shorter LTL. Hence, it remains unclear whether individuals with repeated and extensive exposure to ACEs potentially suffer from accelerated biological aging, as indexed by TL, or not, and to the best of our knowledge, no studies have examined the effects of ACEs on BTL in later life in individuals with and without PTSD.

As evidence for the potential causal role of ACEs in shortening telomeres, some studies have reported a faster rate of decline in TL in children experiencing adversity. In one prospective longitudinal study of 236 children, Shalev and colleagues found that children with more exposure to violence showed significantly more BTL erosion from age 5 to 10 years compared with children with less violence exposure (19). Similarly, Drury and colleagues published a longitudinal study on 136 children living in institutions where buccal swabs were collected between 6 and 30 months of age and compared with BTL from 54 months of age (20). In their results, percentage of time spent in the institution was significantly and negatively associated with BTL and remained so, even after controlling for potential cofounders like gender, ethnicity, low birth weight, and age at telomere collection.

Overall, these studies consistently indicate that both ACEs and PTSD status are associated with shorter TL. However, we do not know if ACEs and PTSD status exert an influence on BTL over the entire lifespan into old age. Kiecolt-Glaser and colleagues found significantly shorter LTL and heightened inflammatory markers in a community sample of *n* = 132 subjects (mean age of 69.70 years, including 58 dementia caregivers and 74 non-caregivers) when participants reported some form of physical, emotional, or sexual abuse during childhood (21), and Savolainen et al. (16) found some evidence indicating that lifetime history of adverse events might be associated with late life LTL in American samples. However, it is not yet clear if this effect is restricted to the immune system (as indexed by changes in leukocytes) or if it will be replicated in a population who experienced such early life adversity as the Verdingkinder, a European population.

Our aim was to explore BTL differences in a sample of former Swiss indentured child laborers, currently of old age and to compare them to a healthy control group. The indentured child laborers in this study experienced both parental and familial separation as well as childhood maltreatment. Both factors were reported to have a negative impact on LTL (15, 17, 19). We examined: (a) BTL in former indentured child laborers reporting childhood adversities but no PTSD symptoms compared with healthy controls; (b) BTL in former indentured child laborers reporting childhood adversities and showing PTSD symptoms compared with healthy controls; and (c) the association between total reported childhood adversities and BTL. Previous studies using this specific sample have shown that some former child laborers screened positive for partial and full-blown PTSD, while others remained relatively unaffected in terms of PTSD psychopathology [e.g., Ref. (3)]. Exposure to ACEs is generally substantially higher for former indentured child labors compared with a community sample of healthy controls recruited for lack of trauma exposure. Based on previous research findings, we predicted that (a) former indentured child labors would have shorter BTL compared with healthy controls; (b) individuals screening positive for PTSD will have shorter BTL compared with participants without PTSD; and (c) more childhood trauma exposure will be associated with shorter BTL (17).

## Materials and Methods

### Sample

This study is a sub-study of a larger project that focused on former Swiss indentured child laborers (2, 3). Here, we focused on a subsample of *n* = 67 indentured child laborers who had buccal cells available from a total sample of 141 individuals. For the present study, participants were recruited *via* advertisements in local and national newspapers and magazines, and *via* specific indentured child laborers societies and associations. The following inclusion criteria were applied: (Swiss-)German speaking; a minimum age of 60 years; at least one experienced period of indentured child labor; and report of at least one traumatic event. In addition, a sample of *n* = 62 demographically similar controls was recruited. Criteria for controls were set similar to those of the former indentured child laborers in order to match the samples on key variables including minimum age of 60 years, Swiss-German speaking and were raised in rural upbringing. Furthermore, controls were raised by biological parents and have not been diagnosed with PTSD or any other psychiatric disorder. The overall study sample included *n* = 129 subjects. Written informed consent was obtained from all participants, and the study was approved by the ethical committee of the Canton of Zurich (Switzerland).

### Procedure

Psychometric data for the “Verdingkind” sample were collected between 2010 and 2012. Participants were asked to respond to a set of standardized questionnaires and structured interviews, including a testing of cognitive capacities within another project [see Ref. (3) for more information]. The interviews lasted between 2 and 3 h and were conducted either at participants’ homes or at the University of Zurich by trained research assistants and doctoral students. In the context of another study, conducted between 2012 and 2013, a subsample of this indentured child laborers donated buccal epithelial cells. Simultaneously, a sample of healthy controls was recruited and screened with the same questionnaires as the Verdingkind sample. Contrary to the Verdingkind sample, the buccal epithelial cells of the controls were collected immediately following the interview in the first semester of 2014. Hence, the two sets of buccal samples (Verdingkind vs. controls) were analyzed separately due to the independent sampling.

### Materials

#### Childhood Trauma Questionnaire – Short Form

The Childhood Trauma Questionnaire – Short Form (CTQ-SF) is a 28-item self-report inventory that provides brief and reliable screening for histories of abuse and neglect. It inquires about five types of maltreatment: emotional, physical, and sexual abuse, and emotional and physical neglect. The CTQ (including CTQ-SF) is one of the most widely used instruments to assess childhood maltreatment and trauma and has been extensively cross-validated (22–24). In the original validation study on a US community sample (*n* = 1,007) aged between 18 and 65 years, a Cronbach’s α of 0.91 was obtained for the total score, 0.85 for emotional neglect, 0.83 for emotional abuse, 0.94 for sexual abuse, 0.69 for physical abuse, and 0.58 for physical neglect (25). In a recent study on 565 Swiss patients with mainly anxiety disorder or depression (86%), pedophilia (3%), or sleep disorders (11%), as well as 86 psychology students, the German questionnaire version obtained Cronbach’s α of 0.82 for physical abuse, 0.83 for emotional abuse, 0.90 for sexual abuse, 0.91 for emotional neglect, and 0.53 for physical neglect (22). Thus, all subscales seem to have acceptable reliability, except physical neglect. Furthermore, cutoff scores for each subscale as well as for the total score have been suggested (i.e., ≥13 for emotional abuse, ≥10 for physical abuse, ≥8 for sexual abuse, ≥15 for emotional neglect, ≥10 for physical neglect, and ≥56 for the total score) (26).

#### Short Screening Scale for PTSD

The short screening scale (SSS) for PTSD is a seven-item instrument designed to screen PTSD symptomatology in trauma survivors. The seven items – five avoidance and numbing items (cluster C) and two hyper arousal items (cluster D) – were taken from the extensively validated posttraumatic stress diagnostic scale. Respondents evaluate for each item how many times it occurred over the last week. All symptoms that occur for more than two times a week add up to the overall test score. The authors suggest a cutoff score of four which best balances the scales sensitivity (80%) – the ability to detect patients with PTSD – and specificity (97%) – the ability to detect patients who do not have PTSD. Furthermore, Maercker and Pielmaier (27) suggest a category of partial PTSD when at least one of the C-cluster symptoms and one of the D-cluster symptoms occurring two times or more per week were present. In a study on an adult German population (28) and a Swiss population [age >65 years (27)], the SSS reached a Cronbach’s α of 0.90 and 0.68, respectively.

#### Covariates

Depressive symptoms were measured using the Geriatric Depression Scale [GDS (29)]. The 15 questionnaire items assesses the presence of depressive symptoms based on a yes–no dichotomous response scale (e.g., “Have you dropped many of your activities and interests?”). In a German sample of 43 hospitalized patients, the GDS reached a Cronbach’s α of 0.91 (30). Physical and mental functioning was assessed with a 12-item version of the Short-Form Health Survey (SF-12) (31). Additional study covariates were age, sex, self-evaluated financial situation (from “poor” to “very good”), and total years of education. Self-evaluated financial situation (i.e., “How would you describe your financial situation right now?”) was used as a proxy for social economic status.

#### Buccal Cell Telomere Length

For BTL, participants were asked to rinse their mouths with water twice for 15 s prior to sample collection using two to four Isohelix Buccal Swabs (Cell Projects, Kent, UK). According to the manufacturer’s instructions, participants were assisted in the sample collection procedure by the research assistant or doctoral students. Each buccal swab was rubbed firmly against the inside of both cheeks for 30–60 s (32, 33). The collected buccal epithelial cells were stored with DNA stabilizer until further preparation. The insertion of a Dri-Capsule (Cell Projects, Kent, UK) allowed the sample to be stored at room temperature without DNA degeneration (34). Buccal cell samples were then sent to North America and analyzed by DNA Genotek (Ottawa, ON, Canada) using quantitative polymerase chain reaction (qPCR) to assess BTL (35, 36). TL was determined using qPCR on a Life Technologies 7900 HT real-time instrument and Life Technologies SDS v2.4 software to estimate absolute TL. Primers were obtained from Integrated DNA Technologies. Briefly, the protocol described by O’Callaghan et al. (35) uses an oligomer standard containing 14 TTAGGG telomeric repeats and a standard curve using a single-copy gene standard (*36B4*) to estimate both the mean TL per reaction and the mean diploid genome copies for each sample. The TL per diploid genome and the length per telomere are then calculated according to methods described by O’Callaghan and colleagues (35). All measurements were repeated in triplicate, and mean results accepted only if the SD of the cycle threshold (*C*_t_) was <1 *C*_t_. Approximately 90% of samples typically pass this quality check (QC) parameter. A sample failed the analysis if it did not pass QC values for the telomere or the single-copy gene assay or both.

### Statistical Analyses

To test whether participating indentured child laborers differed significantly from the rest of the sample, Welch’s two-sample *t*-test was conducted for continuous variables and chi-square tests for categorical variables. Pearson correlations were computed to assess the association between BTL and the other continuous measures used in our study.

Our primary hypothesis was tested using analysis of covariance (ANCOVA) by comparing BTL differences between the former indentured child laborers who screened positively or negatively for full or (partial) PTSD symptomatology and the healthy controls. In these analyses, sex, age, years of education, GDS scores, and physical and mental functioning were adjusted for. *Post hoc* group comparisons were conducted with the Tukey’s honest significance test (HSD) to account for the differences in the group sizes (37).

Since the CTQ yields continuous scores, the two samples (indentured child laborers and healthy controls) were pooled together and a stepwise hierarchical linear regression analysis was performed in three steps with BTL as the outcome variables and CTQ total scores as a predictor. The first step contained the raw CTQ total scores as a predictor. For the second model, we included age as a covariate, since TL is associated with chronological aging. In the third step, we added all of the mentioned covariates (i.e., age, sex, years of education, self-evaluated financial situation, and GDS scores) to the model.

Four controls were excluded from the analyses due to technical failure of the BTL assay. In order to account for leverage of statistical outliers, outliers were removed according to their Cook’s distance (38). Based on a simple model where group-status predicted BTL, a Cook’s distance was computed for every single case. The cutoff was set to 4/*n* (39). Six participants were identified as influential outliers based on Cook’s distance scores and therefore removed from further analyses. After data cleaning including removal of the outliers, visual inspection and Shapiro–Wilks test [*W* = 0.98, *p* = 0.18 (40)] suggested a better approximation of the BTL measurers to normal distribution.

Data handling and all analyses were conducted with R (41).

## Results

### Sample Characteristics

First, we checked whether the indentured child laborers who donated buccal swabs were markedly different from the original sample. Apart from marginally higher levels of emotional (*t*[135.24] = 1.93, *p* = 0.06) and physical abuse (*t*[132.79] = 1.92, *p* = 0.06) on the CTQ, the participating indentured child laborers did not differ significantly from the rest of the (non-participating) indentured child laborers sample (all *p* > 0.16; see Table 1).

**Table 1:** **Sample characteristics of participating Verdingkinder and non-participating Verdingkinder**.

	Participating Verdingkinder	Non-participating Verdingkinder	
		
	M (SD)/*n* (%) *n* = 67	M (SD)/*n* (%) *n* = 74	*p-*Value
**Demographics**			
Females^b^	30 (44.78)	28 (37.84)	0.51
Age^a^	76.33 (6.13)	77.76 (7.34)	0.21
Years of education^a^	10.47 (2.15)	10.22 (3.32)	0.57
Self-evaluated financial situation^b^			0.35
Poor	6 (8.96)	13 (17.57)	
Fair	17 (25.37)	22 (29.73)	
Good	28 (41.79)	25 (33.78)	
Very good	15 (22.39)	13 (17.57)	
Marital status^b^			0.87
Single	3 (4.05)	5 (6.76)	
Married	28 (41.79)	28 (37.84)	
Separated or divorced	17 (25.37)	17 (22.97)	
Widowed	19 (28.36)	24 (32.43)	
GDS scores^a^	3.17 (3.01)	3.88 (4.01)	0.26
SF-12: physical functioning^a^	42.86 (11.55)	42.16 (11.72)	0.72
SF-12: mental functioning^a^	48.31 (10.29)	49.25 (10.71)	0.60
**CTQ scores**			
CTQ total score^a^	74.52 (20.42)	70.00 (17.01)	0.16
Emotional abuse^a^	15.27 (6.14)	13.32 (5.77)	0.06
Physical abuse^a^	14.00 (6.99)	11.85 (6.22)	0.06
Sexual abuse^a^	9.90 (6.59)	8.92 (5.77)	0.35
Emotional neglect^a^	20.40 (5.17)	21.09 (5.05)	0.41
Physical neglect^a^	14.97 (4.03)	15.29 (3.64)	0.63

*Two participants of each Verdingkinder group refused to disclose information about their self-evaluated financial situation*.

*^a^Welch’s two-sample t-test*.

*^b^χ^2^-test*.

The total sample for the present analyses comprised 120 participants, including 62 indentured child laborers and 58 controls. Table 2 provides an overview of the samples’ characteristics. There was no significant difference in gender between the two samples (χ^2^ [1, *n* = 120] = 0.51, *p* > 0.10). The former indentured child laborers were significantly younger than the healthy controls (*t*[119] = 2.15, *p* < 0.001) and further reported significantly fewer years of education (*t*[119] = −5.36, *p* < 0.001), as well as marginally lower financial status (χ^2^[3, *n* = 120] = 8.60, *p* = 0.03). A significant mean difference in years of education is to be expected, since many indentured child laborers were hindered from attaining an education (1). Furthermore, the two samples differed in mental (*t*[119] = −6.13, *p* < 0.001) and physical functioning (*t*[119] = −4.51, *p* < 0.001). As expected, indentured child laborers also showed significantly higher total and subscale CTQ scores (all *t*[119] > 4.85, *p* < 0.001).

**Table 2:** **Sample characteristics of Verdingkinder and controls**.

	Verdingkinder	Controls	
		
	M (SD)/*n* (%) *n* = 62	M (SD)/*n* (%) *n* = 58	*p-*Value
**Demographics**			
Females^b^	27 (43.55)	30 (44.11)	0.48
Age^a^	76.19 (6.18)	71.85 (5.97)	<0.001
Years of education^a^	10.45 (2.16)	13.35 (3.57)	<0.001
Self-evaluated finical situation^b^			0.04
Poor	5 (8.06)	0 (0.00)	
Fair	16 (25.81)	10 (17.24)	
Good	26 (41.94)	37 (63.79)	
Very good	14 (22.58)	11 (18.97)	
Marital status^b^			0.33
Single	2 (3.23)	3 (5.17)	
Married	25 (40.32)	23 (39.66)	
Separated or divorced	17 (27.42)	11 (18.97)	
Widowed	18 (29.03)	12 (20.69)	
GDS scores^a^	3.23 (3.10)	0.45 (0.80)	0.23
SF-12: physical functioning^a^	42.71 (11.91)	50.64 (6.88)	<0.001
SF-12: mental functioning^a^	48.44 (10.37)	56.82 (2.80)	<0.001
**CTQ scores**			
CTQ total score^a^	73.27 (20.17)	34.31 (7.77)	<0.001
Emotional abuse^a^	14.98 (6.18)	6.60 (2.38)	<0.001
Physical abuse^a^	13.58 (6.80)	5.76 (1.90)	<0.001
Sexual abuse^a^	9.66 (6.45)	5.57 (1.51)	<0.001
Emotional neglect^a^	20.12 (5.27)	9.45 (4.21)	<0.001
Physical neglect^a^	14.92 (4.14)	6.93 (2.25)	<0.001

*Sample sizes are indicated for samples after exclusion of outliers*.

*^a^Welch’s two-sample t-test*.

*^b^χ^2^-test*.

Forty-eight of the 62 (77.42%) former indentured child laborers reported CTQ total scores high enough to indicate moderate to severe childhood trauma, while the scores ranged from 32 to 115. The range for CTQ scores in the 58 controls spanned from 25 to 56, with only one control scoring at the clinically significant cutoff of 56 and all others scored 53 or lower. Nine former indentured child laborers screened positive for partial PTSD and 12 for full PTSD. For subsequent analyses, individuals with partial and full PTSD were combined into one group, resulting in 21 individuals reporting previous moderate to severe childhood trauma and PTSD, 41 participants with moderate to severe childhood trauma but no PTSD, and 58 controls reporting neither previous moderate to severe childhood trauma nor PTSD.

### PTSD and BTL

**Figure 1:**
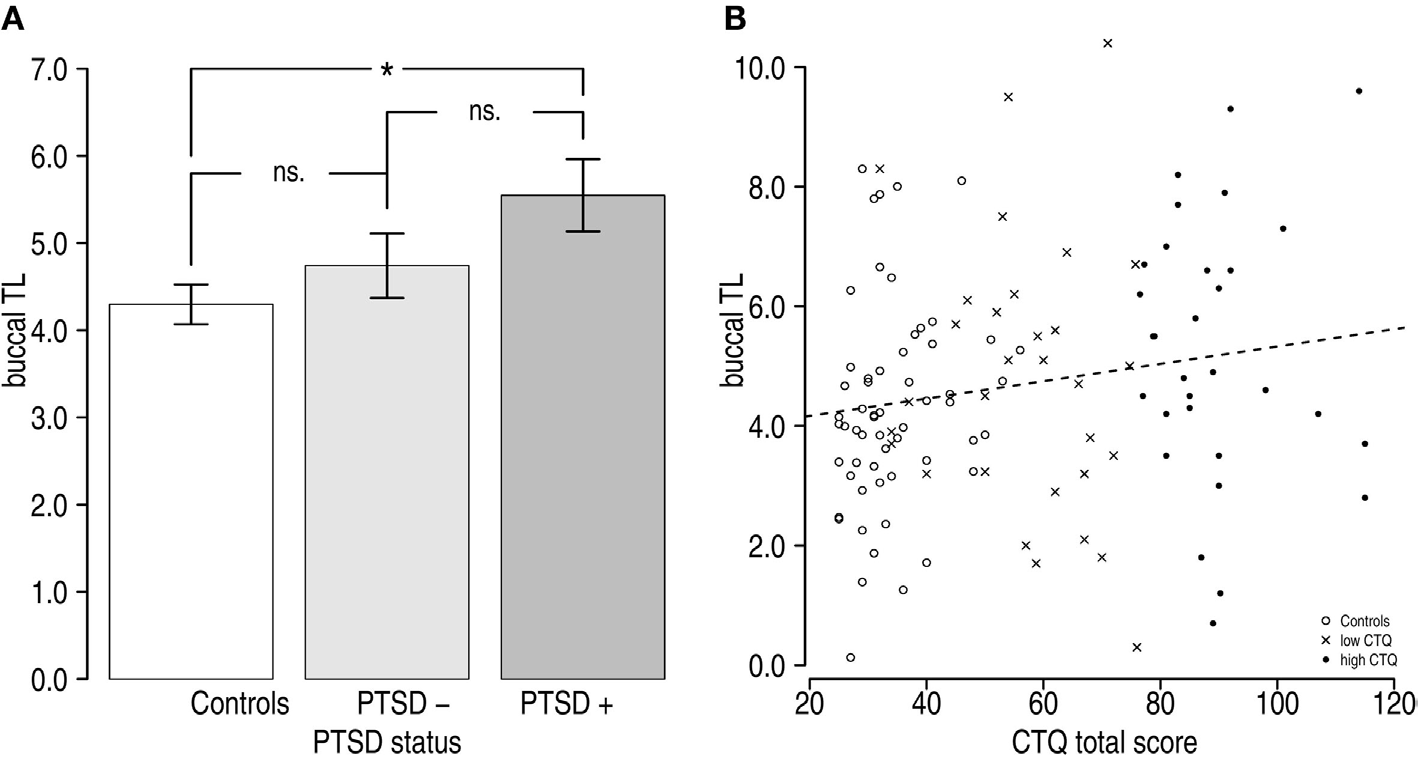
**Buccal telomere length (BTL) differences by group**. **(A)** Analysis of covariance indicated that there was a significant effect of PTSD status [*F*(2,109) = 3.27, *p* = 0.04, η^2^ = 0.06]. BTL tended to increase from controls (*n* = 58, M = 4.30, SD = 1.73) to former indentured child laborers with PTSD (*n* = 21, M = 5.55, SD = 1.90), whereby former indentured child laborers with PTSD negative symptomatology (*n* = 41, M = 4.74, SD = 2.38) did not demonstrate significantly shorter BTL than either indentured child laborers with PTSD positive status or controls (both *p* > 0.292). **(B)** Scatterplot illustrating the relationship between unadjusted BTL and Childhood Trauma Questionnaire total scores. Higher childhood trauma scores were associated with longer mean BTL.

In order to test our hypothesis about the relationship between PTSD symptom status and BTL, ANCOVA including three factors (*controls* vs. *PTSD negative* vs. *PTSD positive*) were conducted by simultaneously controlling for the effects of age, sex, years of education, GDS score, and mental and physical functioning. Analyses indicated that PTSD symptomatology was significantly associated with BTL, *F*(2,109) = 3.27, *p* = 0.04, η^2^ = 0.06 in these analyses. Tukey’s HSD indicated that healthy controls showed significantly (*p* = 0.04) shorter BTL (*n* = 58, M = 4.30, SD = 1.73) compared with the PTSD positive former indentured child laborers (*n* = 21, M = 5.55, SD = 1.90), resulting in a BTL mean difference of 1.25 (95% CI: 0.04–2.46). No significant differences between the PTSD negative former indentured child laborer group and the PTSD positive former indentured child laborer group (*n* = 41, M = 4.74, SD = 2.38), or between the PTSD negative former indentured child laborer group and the control group were detected (both *p* < 0.29, resulting in a BTL mean difference of 0.80 [95% CI: −0.46 to 2.08] and 0.44 [95% CI: −0.53 to 1.41], respectively). Figure 1A provides a visualization of the results.

### ACEs and BTL

Correlation analyses indicated that BTL was only marginally associated with CTQ scores and not associated with age or the other measures in the pooled sample. Furthermore CTQ was associated with all measures, except the participants’ age. Table 3 gives an overview of these correlations results.

**Table 3:** **Correlations between BTL and the continuous covariates**.

Measures	1	2	3	4	5	6
1. BTL	–					
2. CTQ total score	0.18^‡^	–				
3. Age	0.06	0.08	–			
4. Years of education	−0.09	−0.39**	−0.35**	–		
GDS score	0.09	0.51**	0.29**	−0.35**	–	
SF-12: mental functioning	−0.06	−0.49**	−0.12	0.29**	−0.69**	–
SF-12: physical functioning	−0.01	−0.34**	−0.26**	0.14	−0.42**	0.23*

*Correlations were conducted over the sample of n = 120 participants who enrolled in the main analyses*.

*^‡^p > 0.10*.

*p > 0.05.

**p > 0.01.

To further test whether BTL was associated with CTQ scores, we performed a hierarchical linear regression analysis. First, only CTQ scores were entered into the model – again – resulting in a marginally significant effect showing that higher CTQ scores were associated with longer BTL (β = 0.18 [95% CI: 0.00–0.35], *t* = 1.92, *p* = 0.05); see Figure 1B. Since BTL is associated with biological aging, a second model adjusted for age was computed. In this model, age was not associated with BTL (β = 0.05 [95% CI: −0.13 to 0.23], *t* = 0.53, *p* = 0.60), and the association between the CTQ total score and BTL remained marginally significant (β = 0.17 [95% CI: −0.01 to 0.35], *t* = 1.90, *p* = 0.06). In the third and final step, the remaining covariates sex, years of education, GDS score, and mental and physical functioning were included in the model. The fully adjusted effect size of the CTQ total score was slightly larger but remained only marginally significant (β = 0.25 [95% CI: −0.01 to 0.49], *t* = 1.64, *p* = 0.06).

### Within-Group Associations between Trauma and BTL

In exploratory analyses, we examined relationships between CTQ and BTL within the two subsamples of former indentured child laborers (*n* = 62) and the controls (*n* = 58). As with the full sample analysis, we computed three different regression models for both samples. In the first model, we only predicted BTL with CTQ total scores; in the second model, we adjusted for age, and in the third model, we adjusted for all covariates (i.e., sex, years of education, self-evaluated financial situation, and GDS scores).

In these models, there was a trend toward an association between higher CTQ total scores and longer BTL within the former indentured child laborer sample (β = 0.03, *t* = 0.23, *p* = 0.08). However, when adjusted for age alone (β = 0.10, *t* = 0.68, *p* = 0.50) or all of the remaining covariates (β = 0.12, *t* = 0.62, *p* = 0.46), this relationship between CTQ scores and BTL was not present. In the control sample, there were no significant associations between CTQ scores and BTL either in unadjusted models (β = 0.20, *t* = 1.52, *p* = 0.14), models adjusted for age (β = 0.18, *t* = 1.40, *p* = 0.17), or models adjusted for all of the covariates (β = 0.21, *t* = 1.50, *p* = 0.14). Notably, however, even in the control sample, the trend was toward higher CTQ scores being associated with longer BTL.

## Discussion

The aim of this study was to assess the association between PTSD, childhood trauma, and BTL in a sample of elderly formerly indentured Swiss child laborers (i.e., “Verdingkinder”). Contrary to our hypotheses, we were unable to observe shorter mean BTL in the sample of former child laborers compared with a group of healthy control subjects. Instead, former indentured child laborers screening positive for partial or full PTSD symptomatology showed longer BTL compared with healthy controls; this effect remained significant even after controlling for age, sex, years of education, and depression. The differences between the healthy controls and indentured child laborers without significant PTSD symptoms as well as the difference between indentured child laborers with PTSD compared with without PTSD were both statistically non-significant (see Figure 1A). We also examined if self-reported childhood trauma was associated with BTL. Again – contrary to our hypotheses – we found no significant associations and in fact, a tendency for higher CTQ scores to be associated with longer age-adjusted BTL. Thus, we failed to replicate previous findings of shorter TL associated with PTSD and child trauma exposure. Explanations include possible resilience in our group of surviving elderly maltreated indentured child laborers and a lack of persistence of the effects of child trauma into late life.

To the best of our knowledge, this is the first study to examine the association between PTSD symptomatology, ACEs, and BTL in a sample of elderly people. The indentured child laborers in our sample experienced both parental and familial separation as well as high rates of maltreatment. Prior research shows that parental separation in itself is a form of early adversity that can have strong negative impact on mental and physical health (17). Contrary to our hypotheses and in contrast with some studies (16, 17, 19, 21), but in line with recent findings (18), shorter mean BTL was observed in controls compared with indentured child laborers with PTSD. Prior studies in older adults have assessed LTL instead of BTL (16, 17, 19, 21). Given that our results are inconsistent with these prior studies, it is also possible that the effects of early life adversity on cellular aging are not evident in buccal cells.

Alternatively, the finding of longer BTL in traumatized individuals with PTSD symptomatology may suggest a resilience hypothesis. Given the known adverse effects of psychological stress on biological age and mortality, it is possible that less healthy and less resilient individuals from the indentured child laborer population died earlier and never had the chance to participate in this study in the first place. There is currently no specific study that reports on mortality in former indentured child laborers, but other studies do suggest that traumatic stress may lead to early mortality (42, 43). It is therefore possible that those individuals who did participate could be described as a potentially unique selection of resilient survivors who, despite their negative psychological experiences, did not necessarily undergo accelerated biological aging.

In opposition to this hypothesis are the high rates of PTSD symptom scores observed in our sample of indentured child laborers. If our sample is truly resilient, it is surprising that they demonstrate such high levels of trauma-related psychopathology. One explanation for these high levels of trauma-related psychopathology might be found in political issues within Switzerland. Over the last few years, there has been a national debate in Switzerland over the compensation of peoples who have been indentured. It is therefore possible that these factors influenced the participants’ responses such that some participants hoped to increase their chances for compensation when they reported more childhood trauma and demonstrated PTSD symptoms. Such biases may be conscious or unconscious, as indicated by studies of US military veterans (44).

We further tested our *post hoc* assumption that our participants might constitute a sample of particularly resilient subjects by dividing the sample into a group with longer mean BTL and a group with shorter mean BTL by a median split. These analyses did not reveal any significant associations between BTL and any of the variables, including gender, mental or physical functioning, familial status, financial status, or depression symptomatology in any of the two groups. Therefore, the assumption that the surviving elderly indentured child laborers were resilient was not supported. Nevertheless, it is possible that our sample were physiologically resilient to stress, even if not psychologically resilient. Unfortunately, our design did not allow us to control for survivor effects, and thus, this hypothesis must be addressed in a future prospective longitudinal design. And like indicated in Section “Introduction,” there is unfortunately also no public record (e.g., vital records) that could further explore the mortality rate and resiliency of former indentured child laborers in Switzerland and compare it to that of the general Swiss population. Nevertheless, similar findings were reported in another trauma survivor study (45).

Finally, this finding has to be interpreted with caution. First, TL research (16, 17, 21) has predominantly focused on TL derived from leukocyte populations or, in cases where buccal epithelial cells were used, the analyses were conducted in younger samples (19). It has not yet clearly been established how BTL behaves as a function of age, and whether the results can be extrapolated to other tissue samples, such as leukocyte samples, since buccal epithelial cells are exposed to a number of exogenous stressors and have a very prolific nature. So far, stability between different tissue samples remains an ongoing field of research. Only a few studies have compared TL concordance across different tissues (46–51). To the best of our knowledge, there is currently no study that observed TL dynamics across multiple time points from different tissue samples. Nevertheless, some cross-sectional data indicate that there may be substantial synchrony across different tissue samples. For example, did Friedrich and colleagues find that the associations between epidermis TL, LTL, and synovial TL samples were very high in a small sample of nine patients (46). And in two post-mortem samples with 41 and 21 subjects (aged 0–101 years), the correlation between lingual epithelium cell TL and epidermis TL was with *r* = 0.84 and 0.93 also very high (48). Other studies found no significant correlation between BTL and whole blood cell TL in samples with 110 subjects from 18 to 93 years of age (35). Furthermore, while there are several indications that there is a substantial correlation between TL in different tissues, it is important to note that most studies on the subject of tissue-specific TL correlations incorporated only small sample sizes.

### Limitations

This study has some limitations. First, participants were fully aware of their status as indentured child laborers or healthy controls, due to recruitment criteria. For example, healthy controls were excluded when they exhibited psychopathology or when they grew up in problematic households. It is plausible that this recruitment procedure had an indirect effect on the self-reporting tendencies of the participants. Self-reported psychometric data always holds the danger of recall bias. Hence, information about childhood experiences might have been positively (in the case of the control group) or negatively (in the case of the indentured child laborers) biased. Apart from psycho-symptomatology, the groups did also differ in terms of some sociodemographic variables, including age and education.

Second, in order to assess current PTSD symptomatology, the PTSD SSS was used. Despite the instrument’s validity, the SSS can only be regarded as a screening instrument for PTSD symptoms and does not provide clinical diagnosis for PTSD. Third, given the unique sample of former indentured Swiss child laborers, it is not surprising that only few former Verdingkinder participated and also demonstrated current PTSD symptomatology, hence, resulting in a relatively small sample size. Therefore, future studies should aim at incorporating larger samples.

Furthermore, our study had a small sample size, which ultimately limited the statistical power of our study. Sensitivity power analyses indicated that our sample was large enough to detect only medium to large effects. It is therefore worth noting that our study may have lacked sufficient power to detect the effects at hand. However, it is worth noting that the observations in our study went in the opposite direction of our hypotheses. It is therefore plausible that our null findings are not due to a lack of statistical power but rather based on the absence of assumed accelerated cellular aging processes in the collected buccal samples.

Finally, our analyses suffer from a potential batch effect (52). Due to the fact that the control sample was only recruited after the initial indentured child laborers sample, we were not able to simultaneously process both batches. Even though the qPCR protocol is reliable and precautions were taken to ensure validity of the results (35), a potential batch bias is possible. However, even within-group analysis did not indicate confirmation for our general hypothesis (in neither the former child laborer sample nor the control sample), indicating that a lack of a negative association between childhood trauma and BTL cannot be attributed to a batch effect alone. Moreover, higher CTQ scores tended to be associated with longer BTL within the indentured child laborer sample as well as within the full sample. It is also worth noting that secondary analyses revealed no association between the time that passed between BTL and time elapsed between collection of the buccal swabs and the qPCR, indicating that duration of storage did not affect the results. Nevertheless, this methodological issue limits the confidence we can have in our group comparison; however, it is important to note that we also did not support our hypothesis that childhood trauma would be associated with shorter BTL within former indentured child laborers or controls either.

### Conclusion

In conclusion, this is the first study to extend our knowledge about the association between childhood adversity, PTSD, and TL into old age. We were unable to confirm our hypothesis that ACEs and PTSD symptomatology contribute to BTL in later life. Our study results hold the possibility of a resilient-survivor phenotype among populations exposed to severe childhood stress, even though we were not capable of addressing or confirm this alternative assumption in this particular study. Further studies are needed to address this question in a prospective longitudinal manner to account for survivor effects. Given the nature of our findings and lack of other studies on the long-term consequences of childhood adversity on TL biology, the field of stress, and telomere biology should focus more on examining resilience in trauma survivors.

## Author Contributions

AM, AB, and AK designed research; AB and AK performed research; AO contributed consultation and advice; AK analyzed data; AK and AO wrote the earlier versions of the manuscript; and AM and AB revised the manuscript versions.

## Conflict of Interest Statement

The authors declare that the research was conducted in the absence of any commercial or financial relationships that could be construed as a potential conflict of interest. The reviewer AK and handling editor declared their shared affiliation, and the handling editor states that the process nevertheless met the standards of a fair and objective review.
